# The Use of Technology in the Management of Orthodontic Treatment-Related Pain

**DOI:** 10.1155/2021/5512031

**Published:** 2021-03-09

**Authors:** Anand Marya, Adith Venugopal

**Affiliations:** ^1^Department of Orthodontics, University of Puthisastra, Phnom Penh, Cambodia; ^2^Department of Orthodontics, Saveetha Dental College, Saveetha Institute of Medical and Technical Sciences, Chennai, India

## Abstract

Orthodontic pain is one of the negatives associated with fixed orthodontic treatment that cannot be avoided. This pain usually comes around the wire placement period and gradually decreases once the endogenous analgesic mechanisms start functioning. Over the years, several treatment modalities have been utilized for relief from orthodontic pain, and these include mechanical, behavior modification, and pharmacological methods. However, in the last decade, there are several newer methods employing the use of technology that have come up and are being used for alleviating pain. From computerized indirect bonding to virtual treatment planning, technology has slowly become a vital part of an orthodontist's repertoire. The digital age is here, and orthodontics must embrace the use of technology to help improve the quality of life of patients.

## 1. Introduction

Orthodontic pain is one of the negatives associated with fixed orthodontic treatment that cannot be avoided. It is caused by vascular occlusion brought about by orthodontic forces and involves the release of inflammatory mediators that regulate the movement of inflammatory cells around the teeth. This pain usually comes around the wire placement period and gradually decreases once the endogenous analgesic mechanisms start functioning. Over the first 24 hours, this pain is usually found to increase and then taper down within a week after initial bonding [1]. This is an important aspect to consider because over this period, the patient's quality of life is impacted in terms of impaired speech, oral ulcers, difficulty in mastication, tooth mobility, and gum inflammation [2].

Previous research has shown that the periaqueductal grey and dorsal raphe maintain an important role in the management of orthodontic pain. Over the years, several treatment modalities have been utilized for relief from orthodontic pain, and these include mechanical, behavior modification, and pharmacological methods. However, in the last decade, there are several newer methods employing the use of technology that have come up and are being used for alleviating pain. Many of these methods are currently being researched to understand their precise benefits as well as effectiveness in managing pain. Some of the newer pain alleviation techniques are explained in this article.

## 2. Low-Level Laser Therapy (LLT)

This has found considerable use for pain management in both medical and dental fields. This involves the laser irradiation of both the arches using a low-level laser. The expansion for the acronym “laser” is “light amplification by stimulated emission of radiation.” The main point of difference between lasers and other light sources is their coherence which helps narrow their focus to a specific area, even over long distances [3]. There are two types of lasers in use: high-intensity and low-intensity, and these differ in terms of their working action and potency [4]. The low-intensity laser also known as a cold laser does not have any destructive potential and rather stimulates anabolic activities and bone remodeling and enhances tooth movement [5]. Commonly employed low-energy lasers include gallium-arsenide (904 nm wavelength), semiconductor (780–950 nm wavelength), helium-neon (632.8 nm wavelength), and gallium-aluminium-arsenide (805 nm wavelength) [6]. Previous research has shown that the gallium-aluminium-arsenide laser has greater penetration and is therefore more effective in managing pain associated with orthodontic treatment [7–10].

It has been seen that low-level laser therapy induces cellular proliferation which results in differentiation of osteoblasts bringing about bone formation [11–13]. Low-energy lasers have also been found to help enhance orthodontic tooth movement, but more research is still being conducted on the same [14].

### 2.1. Light Emitting Diodes (LEDS)

Photobiomodulation has become popular in recent years, and it involves the use of light-emitting diodes to enhance healing, control inflammation, and reduce pain and discomfort across different scenarios [15]. Generally, when LEDS are applied to human body tissues, they elicit different reactions such as photothermal, photomechanical, and photochemical reactions which result in various effects [16]. LEDs (in the range of 670 nm) have been shown to be highly beneficial in cancer patients for the management of oral mucositis, but the near-infra-red region light (in the range of 850 nm) has been shown to help in release of growth factors and vasodilation, thereby helping promote wound healing [17, 18]. In recent years, there have been studies conducted on the use of LEDS after lower third molar surgical extractions and have delivered promising results by helping to reduce edema, pain, and swelling [19, 20]. In studies involving rats, it has been seen that the use of LED significantly reduces the quantity of osteoclasts in the periodontal ligaments and enhances orthodontic movement of teeth [21]. In the past few years, there have been various studies conducted on the use of LEDS with many conflicting results in terms of findings. As such, further research is needed to help get more predictable and consistent results.

### 2.2. Micropulse Variations

Pain is a complex phenomenon affected by a multitude of factors and is directly influenced by the amount of force applied. For orthodontic tooth movement to occur, there is inflammation in the periodontium caused by forces which results in the release of inflammatory mediators such as histamine, prostaglandins, and serotonin [22]. These inflammatory mediators act on the nerve endings, and the sensation of pain is transmitted to the brain via the neural pathway. Previous studies have shown that vibration decreases the pain originating from the dentoalveolar complex [23]. The rationale behind the use of vibration devices is the application of the gate control theory according to which pain reduction can be achieved by simultaneously activating nerve fibers with nonnoxious stimuli [24]. One device that generates micropulse vibrations and has become popular in recent times is AcceleDent. There have been studies conducted using this device that have demonstrated a reduction in pain following application during orthodontic therapy [25]. Also, clinical trials have shown that there was reduced overall and biting pain on the use of this device which makes it a highly effective means of pain control during fixed orthodontic therapy. However, more studies are required to understand the precise relationship between generating vibrating stimuli and pain control [26].

### 2.3. Transcutaneous Electrical Nerve Stimulation (TENS)

Pain is one of the commonly associated problems with fixed orthodontic treatment, and previous studies have shown that it is one of the major reasons for patients discontinuing their treatment [27]. TENS is a noninvasive, nonpharmacological technique that utilizes electrical stimulation to reduce periodontal pain. Research conducted using TENS therapy has shown that its main action is to block nerve depolarization, thereby impeding the initial neuropeptide release as well as blocking the positive feedback loop [28]. For TENS therapy to work effectively, clinical studies have shown that simulation must be applied to multiple teeth and both the arches [29]. It was also observed that individual teeth required more than 10 seconds of stimulation to achieve the desired result.

### 2.4. Biofeedback Therapy

This method involves the use of electromyography to alter the physiologic reaction of a patient by neuromuscular manipulation [30]. Using electromyography, a patient's biologic reactions can be recorded, analyzed, and even controlled. The patient's reactions are recorded and then converted to auditory or visual, but the patient may try to influence such reactions [31]. Therefore, the patients are provided training to be able to control their reactions and achieve a relaxed status [32]. The recordings are obtained by placement of electrodes on the skin or by insertion of a needle subcutaneously. The placement depends on whether recordings are desired superficially or from a target group of muscles. Tension is usually recorded in microvolts, and the usual range is from 5 to 40 µv, and baseline data must be obtained prior to obtaining situational data [33]. Previous studies have shown that electromyographic feedback does provide significant pain relief, but the extent of relief is much lower when compared to other pharmacological and nonpharmacological interventions [34]. Therefore, further studies are required to gauge the effectiveness of this modality especially in the management of orthodontic movement-related pain.

### 2.5. Low-Intensity Pulsed Ultrasound (LIPUS)

LIPUS is a noninvasive modality that utilizes acoustic pressure waves with frequencies higher than the human threshold [35]. This method is widely utilized in the field of medicine for diagnosis as well as therapeutic purposes. It has been studied to have a biologically healing effect on tissues when the acoustic waves are passed through them [36]. The waves are of an intensity that does not cause tissue destruction or generate heat. LIPUS has been researched for its use as a therapeutic modality, and it was found to have an accelerating effect on healing of bone fractures [37]. There are also studies that have explored the option of using a combination of low-level laser therapy and LIPUS, and the findings suggest that it could prove to be a particularly useful combination [38]. It was seen from the results that LLT stimulated the mitochondria and enhanced the energy cell cycle, while LIPUS initiated functional movements around the cell membrane, thereby resulting in a synergistic action [39]. Their combination improved histological bone formation and accelerated tooth movement which should be of considerable importance in the field of orthodontics. Like other newer methods, LIPUS and its combination with other modalities must be explored further to arrive at a conclusion as to how it can help alleviate orthodontic pain.

### 2.6. Iontophoresis

This is a noninvasive modality that helps deliver charged as well as uncharged drugs across a membrane using electrical currents [40]. This technique relies on various mechanisms such as electrophoresis, electropermeabilization, and electro-osmosis [41]. It relies on the movement of positively charged ions from the anode to the cathode and the movement of negatively charged ions from the cathode to the anode [42]. This has been found to be a particularly useful technique in the field of medicine for delivering ionic drugs. The process involves the placement of a drug-delivering electrode at the site of drug administration and a return electrode on another area of the body to form an electrical circuit. These electrodes are then connected to a device that generates 4 mA current which is sufficient for ionic delivery of drugs [43]. This technique has been utilized in dentistry and oral care, and significant penetration of drugs has been seen in the oral mucosa [44]. Since the current oral drug delivery systems are not very convenient, this method should be researched further to establish a method for relieving pain, resulting from fixed orthodontic therapy.

### 2.7. Virtual Reality

This is the latest example of technology being used to create a realistic appearing simulation. Sensory illusions can be framed to promote behavioral changes in an environment that can be augmented using digital information [45]. Commonly used devices for sensory stimulation are helmets, headphones, and actuators with sensors that can change the simulation depending on the patient response. The major benefit of virtual reality over the other methods is that patients would feel a psychological presence in the simulated environments because of the sensory immersion [46]. Augmented reality can help alleviate pain by distracting the patient's attention away from pain due to orthodontic treatment. There have been studies done using image-based methods for tracking teeth across a video image 47. CT scans were used in these studies to achieve an approximation of the gingival line. Following this, back-projections of older video frames were used to account for bracket positions [47]. Results from these studies have demonstrated the utility of augmented reality for specific orthodontic interventions though much research into this area is still required.

There have also been research studies conducted on the efficacy of virtual reality for helping control dental pain which have demonstrated the presence of analgesic potential. Patients participating in these studies were subjected to various dental procedures in conjunction with virtual reality distractions [48–50]. The patients were found to experience lesser pain on the rating scale when they were distracted virtually. The same patients reported much higher scores on the pain rating scale in the absence of any virtual distractions, thereby showcasing the adjunctive analgesic potential of augmented reality [48, 49]. With the popularity of augmented reality systems growing with each passing day, it is only a matter of time when specific systems dedicated to various painful conditions are developed and put into practice.

### 2.8. Other Less-Explored Technological Methods for Pain Relief

In the current digital age, there are many methods that are being researched for their use in management of pain. There have been studies conducted on the use of SoLux lamps to deliver heat especially in cases with arthropathies and rheumatic diseases, and the results have been promising [51]. Another noninvasive modality that can be explored for the management of orthodontic treatment-related pain is transcranial magnetic stimulation (TMS). Using this method, alternating magnetic fields are passed through the scalp region which stimulates electrical currents in the neurons. In previously conducted studies, low-frequency stimulation has shown significant pain relief in patients with varying degrees of pain [52]. Another similar method to TMS is transcranial direct current stimulation (tDCS) which stimulates the neurons in the cortex using weak electrical current. Its major advantage over its magnetic counterpart is that its stimulators are small making it preferable for home use [53]. Optogenetics is another futuristic method using which pain modulation can be carried out using brain-stimulation. This can enable action on specific groups of cells to help alleviate pain by means of neuromodulator and neuropeptide pathways [54].

## 3. Conclusion

With the advent of technology, the field of orthodontics is slowly but surely treading the digital path. From computerized indirect bonding to virtual treatment planning, technology has slowly become a vital part of an orthodontist's repertoire. The said pain is one of the few negatives that are associated with fixed treatment starting from the placement of separators. Pain management is an immensely difficult task especially because of the complex systems involved in causing pain. What is needed in the future is a combination of technology and therapy that is planned using data from various clinical trials and experiments. The problem with current modalities is that even being effective, they are not based on a precise neurobiological principle. This is one of the reasons why pain management is still an area of concern when it comes to orthodontics specifically and medicine generally. Pain resulting from orthodontic treatment varies from one individual to another, and it would be a good idea to evaluate a patient's general pain susceptibility during initial diagnosis to be able to plan treatment more effectively. The digital age is here, and orthodontics must embrace the use of technology to help improve the quality of life of patients.

## Data Availability

The data used to support the findings of this study are available from the corresponding author upon request.
